# The sustainable health Agenda in the Americas: Pre-pandemic gaps and 2030 estimates of the SDGs indicators

**DOI:** 10.1371/journal.pone.0270301

**Published:** 2022-06-21

**Authors:** Fabrício Silveira, Luísa da Matta Machado Fernandes, Rômulo Paes-Sousa

**Affiliations:** Instituto René Rachou, Fundação Oswaldo Cruz (FIOCRUZ), Minas Gerais, Brazil; University of Waterloo, CANADA

## Abstract

The preliminary assessments of the impact of the COVID-19 pandemic have recently rekindled worries about the feasibility of the Sustainable Development Goals (SDGs). Notwithstanding the concern voiced by key academic and political actors, the actual evidence on the current gaps and distance from the goals is still very much unknown. This study estimates the global evolution curves for each health-related SDGs indicator in the World Health Organization’s SDGs platform. These curves synthesize the transnational trends at play in the evolution of each health-related topic, offering an average global counterfactual to compare with the actual information for each country. The empirical investigation focuses on the American continent, highlighting the health gaps before the COVID-19 outbreak in 33 countries of the region. The study also extrapolates these trends to predict the evolution of the health-related SDGs in each of these countries over the next decade using as the baseline scenario the International Monetary Fund’s economic forecasts. The results show a widening gap in the region, associated with the differential economic capacity of these countries. Some bottlenecks are shared by most countries in the continent, especially in the themes of violence and infectious diseases. The latter is likely to improve faster than other health themes in the next decade, whereas improvements in the theme non-communicable diseases can be more challenging. The findings provide much needed comparative evidence to guide the countries in the region to set priorities and concentrate efforts to accelerate progress in the health-related SDGs.

## 1. Introduction

The COVID-19 pandemic has been a major global setback for the Sustainable Development Goals (SDGs). Both the current and the future health status of the global population and the socioeconomic organization of our society have been profoundly changed by the pandemic outbreak, which quickly became the leading cause of deaths in many countries [1]. Indirect deaths due to the disruption of essential health services–including the shortage of medicines, staff and diagnostics–according to estimates of the World Health Organization, accounted for twice the number of deaths of the disease by June 2021. These have sharply shortened life expectancy at global, regional and national levels [2]. The consequences of COVID-19 nonetheless are much beyond the health sphere. The obstructions in the production and consumption systems caused a global economic recession, resulting in record rates of unemployment, food insecurity and poverty everywhere [3]. Furthermore, the pandemic proved disproportionately worse for vulnerable populations, including the economically disadvantaged, low-skilled laborers, elderly people and those living in crowded dwellings and/or highly agglomerated areas, exacerbating existing vulnerabilities linked to the underlying inequalities [2].

The post-COVID era will certainly be more challenging than anticipated by early 2020 expectations. Even though the long-term impact on morbidity is still unknown, preliminary studies indicate the worsening of several determinants of health, with potential long-lasting effects in the well-being of the world’s population [4, 5]. Among which, there has been a noticeable increase in individual habits that increase the risk of chronic and non-communicable diseases, such as drinking, smoking, physical inactivity, etc. The COVID-19 pandemic has also alarming implications for individual and collective health and emotional and social functioning [6, 7], what is aggravated by current levels of unemployment.

Such preliminary evidence has been raising concerns about the feasibility of the SDGs’ original commitments. Nature’s editorial on 16 July 2020 claimed that the high costs of the targets and the COVID-19 shock made the original commitments unrealistic [8]. Indeed, the pandemic has hindered much of the previous efforts for the SDGs. The 2021 Sustainable Development Report registered, for the first time since the adoption of the SDGs, a decrease in the global SDG index [1], a figure that is likely underestimated, according to the institution itself, due to data gaps and lags in the publication of official statistics everywhere.

Nevertheless, one may agree that the discussion on renegotiating–rather than reinforcing–the SDGs is misplaced. Firstly, because the impact of COVID-19 on the health-related SDGs is still unknown. Secondly, because the attainment of the SDGs global targets could have prevented most of the epidemiological and socioeconomic consequences of the current crisis [9, 10], which makes the 2030 Agenda into a reference for the post-COVID-19 rebuild. Indeed, as proposed by The Lancet Public Health’s editorial in August 2020, the intersecting challenges of health and sustainability underscored by the pandemics create an opportunity not to be missed in realigning the society around the transformative vision of the SDGs. The COVID-19 outbreak can placed thus as a turning point for the sustainability agenda.

This paper seeks to assess the pre-pandemics health gaps in the Americas and forecast the post-COVID-19 evolution of the health-related indicators for the SDGs in the region. For that, the study proposes a straightforward methodology for identifying the global trends in the SDGs health topics. The approach takes advantage of the increasing availability of comparable international data to offer a global counterfactual that enables estimates to virtually all countries and indicators, even those with limited temporal information.

Therefore, the study provides much needed context for the [re]assessment of the feasibility of the original 2030 Agenda commitments in the region, especially because the only multinational reference for the evolution of the health-related indicators is currently outdated [11]. As of June 2021, the American region accounted for 32% of the COVID-19 deaths worldwide–four times the region’s global population share of 8.4% [2]. The Structural gaps have exacerbated the adverse effects of the pandemic and the Latin-American and Caribbean countries felt an even stronger blow. The ensuing recession has pushed a large share of the local population into extreme poverty and shrunk government resources available for spending on achieving the SDGs, with great disparities across the countries [12].

The next section introduces the methods and data. The results are presented next and highlight the main global trends in the health-related indicators, pre-pandemic gaps in the American countries and the hypothetical progress in the baseline scenario. The discussion reinforces some regional discrepancies in the evolution of the health SDGs. The cross-national approach offers important lessons to explore the local specificities and/or explore intraregional trends, providing guidance for the reconstruction that many countries in the region will have to undergo in the next years.

## 2. Methods and data

The SDGs form an interconnected network of 17 goals and 169 targets monitored by 231 globally-harmonized indicators. Health and wellbeing are explicitly represented in the 13 targets and 27 official indicators of SDG 3, even though the so-called health-related SDGs can comprise a much broader set of indicators scattered across 8 different SDGs [13]. In this study, we adopt the indicators and thematic classification proposed by the World Health Organization, the custodian of the SDGs’ health indicators [14]. Out of the 42 indicators in the institution’s database, we focus on 38. The indicators and the seven health themes are illustrated in Table 1 below.

**Table 1:** WHO’ health-related indicators and global coverage: Summary data availability, by health theme.

Theme	Indicator		Coverage		
			Countries^1^	initial year^2^	end year^3^	
Maternal and reproductive health	311	Maternal mortality ratio (per 100 000 live births)	179	1990	2018
	312	Births attended by skilled health personnel (%)	123	1998	2018
	371	Women of reproductive age (aged 15–49 years) who have their need for family planning satisfied with modern methods (%)	54	2000	2018
	372	Adolescent birth rate (per 1000 women aged 15–19 years)	66	2007	2016
Newborn and child health	221	Children aged <5 years stunted (% height-for-age <-2 SD)	46	1982	2017
	222a	Children aged <5 years wasted (% weight-for-height <-2 SD)	46	1982	2017
	222b	Children aged <5 years overweight (% weight-for-height >+2 SD)	46	1982	2017
	222c	Children aged <5 years underweight (% weight-for-age <-2 SD) (%)	44	1982	2017
	321	Under-five mortality rate (probability of dying by age 5 per 1000 live births)	188	1990	2017
	322	Neonatal mortality rate (per 1000 live births)	188	1990	2017
	3b1a	Diphtheria tetanus toxoid and pertussis (DTP3) immunization coverage among 1-year	188	2016	2017
	3b1b	Measles-containing-vaccine second-dose (MCV2) immunization coverage by the nation	158	2016	2017
	3b1c	Pneumococcal conjugate vaccines (PCV3) immunization coverage among 1-year-olds	132	2016	2017
Infectious diseases	331	New HIV infections (per 1000 uninfected population)	129	2017	2017
	332	Incidence of tuberculosis (per 100 000 population per year)	188	2010	2017
	333	Malaria incidence (per 1 000 population at risk)	106	2010	2017
	334	Hepatitis B surface antigen (HBsAg) prevalence among children under 5 years (%)	188	2015	2015
	335	Reported number of people requiring interventions against NTDs	188	2010	2017
Non-communicable diseases	341	Probability (%) of dying between age 30 and exact age 70 from any of cardiovascular disease, cancer, diabetes, or chronic respiratory disease	181	2000	2016
	342	Crude suicide rates (per 100 000 population)	181	2000	2016
	352	Total (recorded + unrecorded) alcohol per capita (15+) consumption	184	2016	2016
	3a1	Age-standardized prevalence of current tobacco smoking among persons aged 15 years and older	142	2016	2016
Injuries and violence	361	Estimated road traffic death rate (per 100 000 population)	172	2016	2016
	1611	Estimates of rates of homicides per 100 000 population	181	2000	2016
Environmental risks	391	Ambient and household air pollution attributable death rate (per 100 000 population)	181	2016	2016
	392	Mortality rate attributed to exposure to unsafe WASH services (per 100 000 population)	181	2016	2016
	393	Mortality rate attributed to unintentional poisoning (per 100 000 population)	181	2000	2016
	611	Population using at least basic drinking-water services (%)	186	2000	2015
	621	Population using at least basic sanitation services (%)	186	2000	2015
	712	Proportion of population with primary reliance on clean fuels and technologies	185	2000	2016
	1162	Concentrations of fine particulate matter (PM2.5)	186	2016	2016
Health system coverage	381	Universal health coverage (UHC) service coverage index	180	2000	2018
	3c1a	Medical doctors (per 10 000 population)	142	2007	2018
	3c1b	Nursing and midwifery personnel (per 10 000 population)	142	2007	2018
	3c1c	Dentists (per 10 000 population)	115	2007	2018
	3c1d	Pharmacists (per 10 000 population)	107	2007	2018
	3d1	IHR capacity and health emergency preparedness (Average of 13 International Health Regulations core capacity scores, SPAR version)	186	2010	2018
	17192	Completeness of cause-of-death data (%)	81	2009	2017

Notes

^1^ Number of countries with at least one post-2015 data entry

^2^ Initial year of the series

^3^ End year of the series. The temporal availability of data varies with the country. The dictionary of the indicators and complete information on each country’s temporal availability can be found at the institution’s platform for the SDGs. The analysis excluded the following indicators found in the WHO’s database: 3.b.2 - Development assistance for health; 13.1.1—Mortality due to disasters; and 16.1.2—Mortality due to conflicts. The indicator 1.a2—Domestic general government health expenditure (GGHE-D) as percentage of general 185 government expenditure (GGE) (%) was adopted as a co-variate in the study. See <https://www.who.int/data/gho/data/themes/sustainable-development-goals?lang=en>.

Source: WHO. Available at <http://apps.who.int/gho/data/node.sdg.tp-1?lang=em>. Access in 19/04/21

The number of countries with at least one post-2015 data point in the indicator, illustrated in Table 1, evidences the global discrepancies in the coverage of the SDGs indicators. Indeed, some countries may choose not to monitor all targets due to their local specificities, while others may lack the means to provide estimates for most indicators. For instance, while 188 countries reported the neonatal and children mortality statistics (indicators 321 and 322), less than ¼ of them (44 countries) presented statistics for children’s underweight indicator (222c).

The coverage is also very unbalanced across time. Whereas some indicators are long-established in the global health statistical system, others have only recently been harmonized for monitoring the SDGs and, thus, yet depend on adaptations in the usual data collection in most countries [13]. Since longer time series are key to explaining the country-specific phenomena, the application of traditional forecasting methods to evaluate progress in the SDGs can be challenging for some countries and indicators.

In this paper we take advantage of the increasing availability of comparable multinational information and apply panel data and cross-sectional regression methods to estimate the global counterfactual evolution of each of the health-related indicators presented in Table 1. The counterfactual consists of expected level of the indicator at the country’s level of GDP, according to the international evidence. This approach circumvents the lack of temporal data in the analysis and enables–with some additional assumptions regarding the specific health phenomenon and future economic scenarios–the extrapolation of emergent global trends in each indicator to explain the country-specific phenomenon.

The successful estimation of these global evolution curves depends chiefly on the identification of an exogenous variable to represent the level of development, i.e., a summary-variable intended to discriminate between countries in different ‘stages of development’. In this study we adopt the World Bank’s composite measure of Gross Domestic Product (GDP) per capita as such variable. The measure is widely accepted as a summary of key dimensions of the development process, acting either as a determinant or outcome variable [15, 16]. Furthermore, preliminary correlation analyses also revealed a strong association (>0.5) of the variable with most health-related indicators.

In order to avoid a bias in the parameters due to the influence of omitted or other confounding factors, two factors, usually associated with the determination of the health phenomena, were included in the estimated models: the national public health expenditure (% of GDP), also present in the WHO’s database, and the Gini index, published by the World Bank. The first act as a proxy for the availability of health services and the second as a measure of access, since income inequalities are often associated with health disparities within and across countries. Their omission could, potentially, channel the influence of each of these factors over the estimated parameters through the explanatory variable.

To keep the symmetry in the analysis of the health-related indicators, we adopted the same model in all estimations (see Eq 1). Nevertheless, we tested different specifications of the regression model, including specifications with non-linear parameters and the lagged endogenous variable amongst the controls. Indeed, preliminary tests revealed important non-linearities in the global curves, which was corrected by including regional dummies and other co-variates (see below). The lagged endogenous variable, in addition, was adopted in some preliminary specifications to assess the phenomenon’s dependence on initial conditions. In such cases, dynamic panel data models were adopted to avoid endogeneity bias [17]. The results were, in general, non-significant.

$${ln(SDG)}_{i,t}=\propto +\beta_{1}{\ln\left({GDP}_{pc}\right)}_{i,t}+\beta_{2}{health}_{i,t}+\beta_{3}{gini}_{i,t}+\beta_{4}D+f_{i,t}+\varepsilon_{i,t}$$ (1)

where ∝ is a constant, *β* the estimated elasticities, and *f* and *ε* are fixed-effects and the error term, respectively. The subscripts i and t represent the countries and time, respectively, and D is a vector with dummies. The log-log specification in all estimations renders a coefficient that can be read as the percentage change in the indicator for a given percentage change in GDPpc.

As shown, the final specification also included regional dummies to capture the influence of geographic factors on the evolution of the indicators. This is of special importance in this study since the preliminary analysis using partial residual analysis for segments of the global curve in different levels of income confirmed significant non-linearities in the evolution of the health-related indicators. That is, the pace of progress of most SDGs were found to alter with the overall level of GDPpc. For instance, using a more parsimonious model (with no co-variates and controls) in estimations for the World Bank’s four income groups, the estimated *β* showed that although mortality and violence indicators generally showed a negative correlation with GDPpc, progress due to increases in the latter over premature mortality from non-communicable diseases (indicator 341) and homicides (indicator 1611) were 2 and 3 times faster, respectively, in high-income countries, when compared to low-income countries. On the other extreme, changes in maternal mortality (indicator 311) and family planning (indicator 371) due to changes in the GDPpc were stronger in the lowest income ranges. Others, such as children nutrition indicators, showed an anomalous behavior, with inverted signs in different strands of the curve (GDPpc range). The likelihood ratio tests applied to linear and spline models also confirmed the above, but the inclusion of regional dummies and the other co-variates eliminated the significance of the non-linear terms in the final specification.

Preliminary tests with a more parsimonious model (free of controls and co-variates) attested the significance of GDPpc in most health topics. Moreover, the R^2^ test also indicated that a considerable share of the cross-national (and, in some cases, also the temporal) variance of these indicators were explained by the GDPpc. The consistency of the results in different specifications and estimation methods also indicate that the GDPpc was sufficient to limit the noise of other important omitted factors in the association.

Due to temporal and cross-sectional unbalances in the health-related indicators’ series, different estimation methods were applied. Whenever the historical information for the indicator was available, panel data estimation methods were adopted, enabling the identification of fixed-effects of time and space (countries) [18]. This was the case for all indicators inherited from the Millennium Development Goals (MDGs), for which the global harmonization of the datasets begun in the year 2000. Five-year period averages were used to increase the degrees of freedom in the estimation and reduce the serial correlation identified in most series. For the newest indicators, introduced with the SDGs, robust cross-sectional Ordinary Least Squares estimations were adopted.

Whenever statistically significant, the resulting curves enable estimates of the current gap for each health topic. In this study the current gap was measured as the difference between the average post-2015 value of each health-related indicator and its estimated counterfactual (%). Hence, values close to zero indicate that the country is close to the expected value of the indicator, whereas positive/negative values indicate better/worse-off situations, respectively, as a proportion of the counterfactual. A gap of -0.3, for instance, indicates that the country is 30% worse than expected. Likewise, a value of +0.5 indicates that the country is 50% better than expected. The index was adjusted to indicate improvement upwards and worsening downwards regardless of the direction of improvement of the indicator. E.g., mortality and health coverage indicators show improvement in opposite directions, decreasing in the former (<) and increasing (>) in the last.

**Fig 1:**
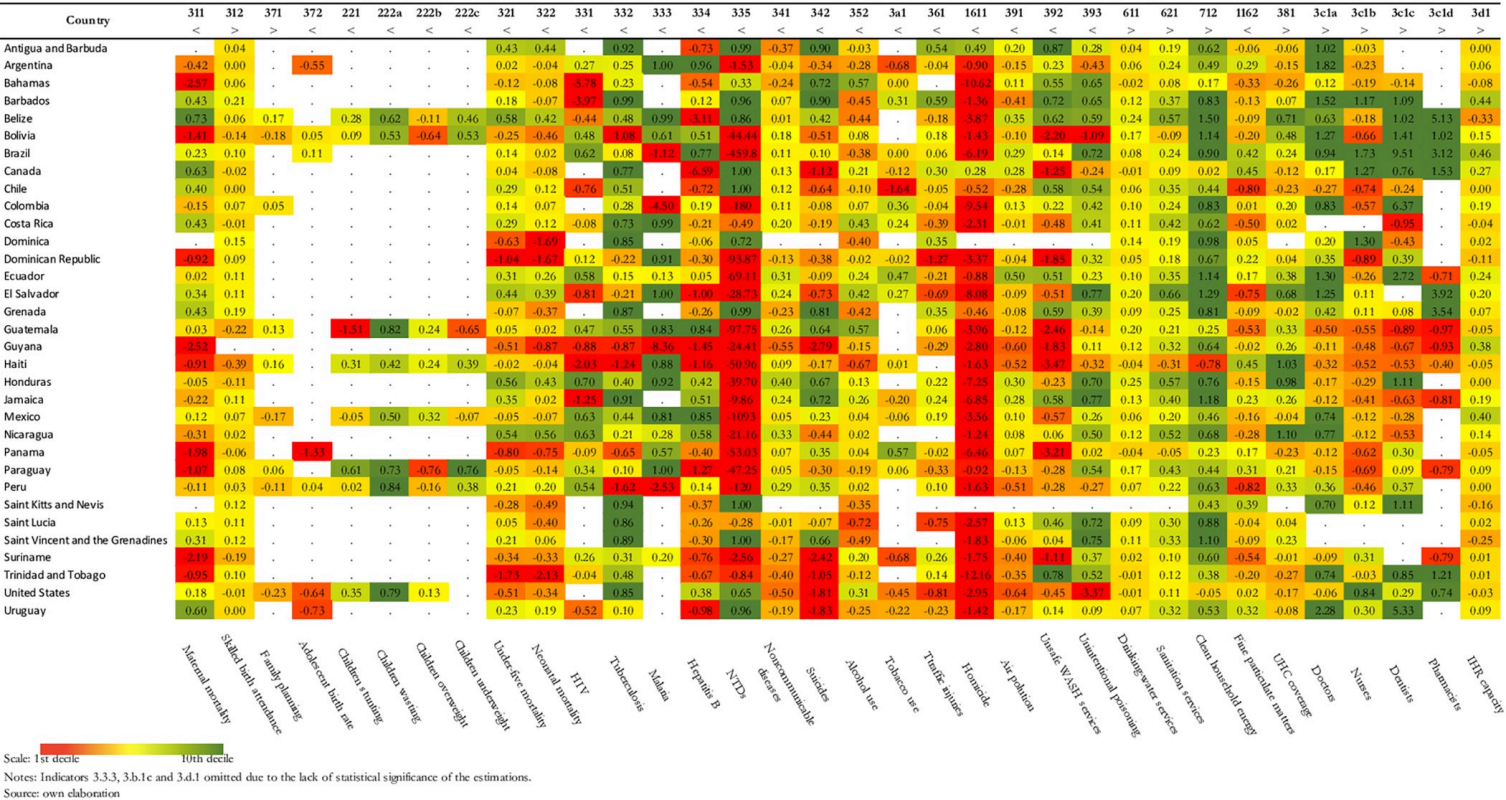
Current health gap scorecard: American countries. Notes: Indicators 3.b.1a,b,c and 17.19.2 omitted due to the lack of statistical significance of the estimations. The gap index was adjusted to indicate improvement upwards and worsening downwards regardless of the direction of improvement of the indicator. For example, mortality and health coverage indicators show improvement in opposite directions (indicated in the first column of the table), decreasing in the former (<) and increasing (>) in the last. Source: own elaboration.

The study also provides 2030 forecasts for the 34 statistically robust health-related series in the 33 American countries of the sample (see Fig 1). These were generated by the extrapolation of the international trends expressed in these curves using the IMF’s revised annual GDP growth projections published in May 2021 [3], considering the country’s initial condition, which compensates for the local specificities not captured in the global curves. The exercise also assumes no significant changes in the level of health investment and in the Gini index in the decade ahead. It is important to mention that these growth projections accounted for the expected economic impact of COVID-19 in the coming years, which allows better calibrated estimates of the evolution of these indicators. We chose the GDP growth forecasts from the IMF for their long-established methodology, credibility and cross-national and time span of the GDP series.

To help visualizing the results of the forecasting exercise the study also provides a measure of projected progress by health theme. The progress was calculated as the sum of the projected gain throughout the decade for the indicators comprised in each health theme (see Table 1) weighted by the number of indicators in the thematic group. As such, the lack of data for any indicator results in a proportional reduction in the total gain measured. The results are adjusted for the direction of the indicator’s progress, since some indicators are worse off increasing, as morbimortality rates, while others are better off increasing, such as health-system coverage.

## 3. Results

Table 2 illustrates the global development curves for the health-related indicators assessed in the chosen specification. Overall, the results indicate the robustness of the estimations, supporting the statistical validity of the inference based in most evolution curves. Both signals and robustness measures are within expectations in most cases (exceptions are discussed below). The R^2^ statistic is also an indicative that most of the cross-country and temporal variance of these indicators are explained by the variables in the model.

**Table 2:** Regression results: Health-related SDG indicators’ evolution curves.

WHO theme	Indicator	log (GDPpc)	Gini index	Health expenditure (% GDP)	Regional Dummies (WHO global regions) (1)					Constant	N	r2	rmse	corr (2)	F
					AMR	EMR	EUR	SEAR	WPR						
Maternal and reproductive health	ind311	-1.0502***	0.00	0.00	-	-	-	-	-	13.6981***	230	0.67	0.16	0.36	39.28
	ind312	0.1183***	0.00	0.01	0.05	0.04	0.14	0.12	0.1634*	3.0204***	152	0.49	0.20	-0.55	14.62
	ind371	0.5865***	0.00	-0.01	-	-	-	-	-	-0.90	204	0.34	0.14	-0.11	5.72
	ind372	-0.5465***	0.01	-0.0332*	0.5462*	-0.33	-0.46	-0.29	-0.6928**	8.6571***	116	0.78	0.55	-0.45	65.14
Newborn and child health	ind221	-0.6931***	0.0163**	-0.01	-	-	-	-	-	8.4202***	262	0.46	0.14		19.36
	ind222a	-0.2507***	0.00	-0.0482***	-1.0411***	0.18	-0.26	0.6862***	-0.17	4.2305***	257	0.68	0.54		78.62
	ind222b	0.2046***	0.0203**	0.00	0.00	0.3786*	0.7839***	-0.4918*	-0.13	-0.9425*	252	0.35	0.59		15.72
	ind222c	-0.6239***	0.0154*	-0.0471***	-0.4731***	0.11	-0.5951***	0.9175***	-0.01	7.4235***	259	0.73	0.56		106.82
	ind321	-1.1218***	0.00	-0.0086**	-	-	-	-	-	13.2557***	492	0.72	0.12		127.73
	ind322	-0.8628***	0.0062*	-0.0124***	-	-	-	-	-	10.1130***	492	0.68	0.11	-0.28	89.38
	ind3b1a	0.03	0.00	0.0093**	-0.02	0.06	0.06	0.12	0.11	3.8931***	112	0.19	0.18	0.10	2.14
	ind3b1b	0.00	-0.01	0.01	0.16	0.14	0.17	0.20	0.2593*	4.4181***	98	0.32	0.20	0.05	3.36
	ind3b1c	-0.06	0.01	0.0161*	0.01	0.17	0.20	0.02	-0.40	4.4335***	81	0.26	0.26		1.09
Infectious diseases	ind331	-0.10	0.0698**	-0.01	-1.8729***	-2.1601***	-1.9050***	-2.7286**	-2.4582***	-2.02	83	0.68	0.98		23.16
	ind332	-0.8926***	0.0089*	0.00	-	-	-	-	-	11.8466***	245	0.35	0.08		20.33
	ind333	-2.1046*	-0.01	-0.1577*	-	-	-0.9148*	-	-	22.0818**	114	0.32	0.33		6.85
	ind334	-0.5736***	-0.03	-0.0604*	-0.9926*	-1.3286*	-3.1261**	-0.6868*	-0.24	7.1719***	112	0.57	0.98		19.54
	ind335	-2.3991***	0.1477***	-0.1396*	1.7390*	0.28		2.9886**	0.39	28.9117***	235	0.75	3.03		131.05
Non-comunicable diseases	ind341	-0.2970***	0.0040*	-0.0130***	-	-	-	-	-	5.6791***	490	0.48	0.07	-0.62	72.55
	ind342	-0.2086**	0.00	0.00	-	-	-	-	-	4.1258***	490	0.09	0.12	-0.64	4.93
	ind352	0.2479**	-0.01	0.01	-0.24	-2.2829***	-0.17	-1.2334**	-0.49	0.14	110	0.61	0.51	-0.23	20.66
	ind3a1	0.01	0.00	0.00	0.07	0.46	0.8398***	0.6524**	0.7373***	2.2108**	100	0.51	0.38		9.47
Injuries and violence	ind361	-0.2373***	0.0192**	-0.0356*	0.11	0.11	-0.4428**	-0.38	-0.07	4.6921***	109	0.73	0.39		63.68
	ind1611	-0.6620***	-0.01	-0.0154***	-	-	-	-	-	8.2065***	490	0.25	0.21		27.58
Environmental risks	ind391	-0.3626***	0.01	-0.0316**	-0.10	0.14	0.5936**	0.36	0.38	7.2567***	112	0.56	0.42		18.31
	ind392	-0.8978***	0.03	-0.01	-1.8525***	-1.08	-2.6747***	-0.82	-1.4430**	9.4696***	112	0.86	0.84	-0.17	117.67
	ind393	-0.8568***	0.0138**	-0.01	-	-	-	-	-	7.2751***	489	0.46	0.17	0.30	33.03
	ind611	0.2190***	0.00	-0.0039**	-	-	-	-	-	2.3409***	490	0.44	0.04	0.47	18.49
	ind621	0.3421***	0.00	-0.0061***	-	-	-	-	-	1.02	491	0.32	0.08	0.06	16.34
	ind712	0.5238***	0.00	0.00	-	-	-	-	-	-1.08	487	0.32	0.12	-0.95	16.74
Health system coverage	ind1162	-0.1882***	0.00	-0.0334***	-0.04	0.8237***	0.01	0.15	0.19	4.9046***	112	0.59	0.36		20.78
	ind381	0.7602***	0.00	0.00	-	-	-	-	-	-2.8643***	487	0.66	0.09		94.15
	ind3c1a	0.8820***	0.00	-0.02	-	-	-	-	-	-6.0251***	291	0.19	0.16	0.55	11.78
	ind3c1b	0.4934**	0.00	0.00	-	-	-	-	-	-1.44	294	0.05	0.20	0.09	3.04
	ind3c1c	1.3176***	-0.01	-0.02	-	-	-	-	-	-11.3952**	250	0.13	0.32	-0.11	5.95
	ind3c1d	1.5310***	0.01	-0.02	-	-	-	-	-	-13.9894**	226	0.18	0.29	-0.88	5.08
	ind3d1	0.5413***	-0.01	0.00	-	-	-	-	-	-0.65	240	0.14	0.09		7.72
	ind17192	0.14	-0.01	0.02	-0.19	-0.5265*	-0.09	-0.76	-0.08	3.2379***	77	0.41	0.33		2.04

Abreviations

*** 0.001 significance

** 0.01 significance

* 0.05 significance.

Regional Dummies: AMR—Americas; EMR—Eastern Mediterranean Region; EUR—Europe; SEAR—South-East Asia Region; WPR—Western Pacific Region.

Notes: The model estimated is shown in Eq 1. (1) A dash (-) indicates the omission of the dummy in the final specification due to collinearity; (2) Only available for panel data estimations. Due to temporal and cross-sectional unbalances in the health-related indicators’ series, different estimation methods were applied. Whenever the historical information was available, panel data estimation methods were adopted, enabling the identification of fixed-effects of time and units [18]. Five-year period averages were used to increase the degrees of freedom in the estimation and reduce the serial correlation identified in most series. For the newest indicators, introduced with the SDGs, ordinary cross-sectional Ordinary Least Squares estimations were adopted.

Source: own elaboration

The results for the co-variates also illustrate important differences in the health phenomena represented by each indicator. The regional dummies were omitted in 19 of the indicators due to collinearity. For these, one might say that either global and/or local factors explain most of the phenomenon. The Gini index was significant in only 10 series. The economic inequality seems to be especially important for explaining infectious diseases and newborn and children health. Signs are all within expectations. Finally, the health expenditure parameter was close to zero in all estimations, but still statistically significant in 17 of the series.

More importantly for the aims of this study, the association between GDPpc and the health-related indicators was significant in roughly 32 of the 38 series. As a general rule, the more elastic the parameter of the association (log GDPpc), the easier is the ‘natural’ progress of the topic. For instance, the parameter of -2.39 of indicator 3.3.5 indicate that for each 1% gain in GDPpc, the international experience indicates a reduction of more than 2.3% in the reported number of people requiring intervention for NTDs. Malaria incidence (3.3.3), with a slope higher than of -2, and neonatal (3.2.1) and maternal (3.1.1) mortality, as well as the number of dentists (3.c.1c) and pharmacists per capita (3.c.1d), with parameters close to 1 in absolute terms, are the other health topics with eased natural progression. On the other hand, the closer to zero the parameter (and/or its statistical insignificance) the costlier the natural progression, which will depend on factors other than the overall socioeconomic development of these countries.

In common, the six indicators with insignificant results for the GDPpc parameter are those with the lowest coverage (N), which partially explains the worse results. This is especially important in the case of completeness of cause-of-death data (17.19.2), for which the sample contained only 77 observations. One may also highlight the case of the 3 children’s vaccine coverage indicators. The estimations for DTP3 (3.b.1a), MCV2 (3.b.1b) and PCV3 (3.b.1c) were all non-significant, with all parameters close or statistically indistinct from zero, an indication that the events associated with the phenomenon have strong country-specific determinants. The last two cases–HIV infections (3.3.1) and Tobacco smoking prevalence (3.a1)–are exceptions within the exceptions. For the former, the F statistic and all regional dummies and the Gini index parameters are robust, an indication that infections by the disease have strong regional determinants and is also influenced by the level of inequality. In the case of the latter, the regional dummies alone explain circa 50% of the phenomenon.

Fig 1 depicts the gap index for the sample of American countries. The color scheme helps illustrating the results. The relative abundance of red areas compared to the green ones shows that the countries in the region were lagging behind expectations even before the COVID-19 crisis. The color pattern in Fig 1 also helps identifying the main bottlenecks of the region: homicides (16.11), neglected tropical diseases (3.3.5), unsafe WASH services (3.9.2) and traffic injuries (3.6.1). Most countries are also underperforming in maternal, neonatal and children mortality. Adding to this negative picture, information on reproductive and children health indicators were scarce in the region.

In contrast, most American countries seem to perform well in indicators of Tuberculosis (3.3.2) and Malaria (3.3.3) incidence; and in indicators of environmental risks, with the exception of mortality due to exposure to unsafe WASH services (3.9.2) and air pollution (3.9.1). Also, virtually all countries are within expectations in the topics of drinking water services (6.1.1), IHR capacity (3.d.1) and skilled birth attendance (3.1.2).

Tables A1a, A1b and A1c in S1 Appendix present the 2030 forecasts for each of the countries in the sample. The predictions are available for all the 32 series with a significant GDPpc parameter, the variable upon which the future scenario is set. To help visualizing the main results, Table 3 offers a summary of the projected progress in each of the seven health themes for the countries and subregions of the continent.

**Table 3:** Projected progress in the health-related themes until 2030: American countries.

Country	Income group*	GDP per capita 2019**	Estimated GDP per capita 2030***	Total estimated growth (2020–2030)***	Average progress in the health-related themes						
					Maternal and reproductive health	Newborn and child health	Infectious diseases	Non-communicable diseases	Injuries and violence	Environ-mental risks	Health system coverage
Antigua and Barbuda	Hi	20115.88	22661.78	12.7%	4.7%	4.1%	7.6%	0.5%	4.1%	5.3%	4.8%
Bahamas	Hi	36973.56	38530.96	4.2%	1.4%	1.5%	2.7%	0.2%	1.5%	1.8%	1.2%
Barbados	Hi	15681.96	16760.34	6.9%	3.0%	2.3%	4.3%	0.3%	2.3%	3.0%	2.6%
Saint Kitts and Nevis	Hi	25277.97	29347.63	16.1%	4.8%	5.1%	9.3%	0.7%	5.2%	6.6%	5.6%
Trinidad and Tobago	Hi	27231.96	27355.31	0.5%	0.2%	0.2%	0.3%	0.0%	0.2%	0.2%	0.2%
Dominica	Up-Mid	12362.03	17114.54	38.4%	14.4%	10.3%	18.4%	1.3%	10.9%	13.9%	14.0%
Dominican Republic	Up-Mid	17027.93	28541.57	67.6%	18.2%	15.3%	26.5%	1.8%	16.6%	21.4%	23.5%
Grenada	Up-Mid	15952.84	20245.94	26.9%	10.7%	7.8%	14.1%	1.0%	8.1%	10.4%	10.0%
Jamaica	Up-Mid	10266.34	11692.23	13.9%	5.9%	4.4%	8.2%	0.6%	4.5%	5.8%	5.3%
Saint Lucia	Up-Mid	15018.53	17174.26	14.4%	6.1%	4.6%	8.4%	0.6%	4.7%	5.9%	5.4%
Saint Vincent and the Grenadines	Up-Mid	12156.94	16150.12	32.8%	12.7%	9.2%	16.4%	1.2%	9.6%	12.2%	12.0%
Haiti	Low	3063.86	2950.10	-3.7%	-1.8%	-1.4%	-2.6%	-0.2%	-1.4%	-1.7%	-1.5%
**The Caribbean**	** **	** **	** **	**19.2%**	**6.7%**	**5.3%**	**9.5%**	**0.7%**	**5.5%**	**7.1%**	**6.9%**
Belize	Up-Mid	7048.34	6951.27	-1.4%	-0.6%	-0.5%	-0.9%	-0.1%	-0.5%	-0.6%	-0.5%
Costa Rica	Up-Mid	19363.11	25003.16	29.1%	9.5%	8.3%	15.0%	1.1%	8.7%	11.1%	10.7%
Panama	Up-Mid	29843.23	41160.17	37.9%	8.9%	10.2%	18.2%	1.3%	10.7%	13.0%	10.8%
El Salvador	Low-Mid	8380.18	10354.98	23.6%	9.5%	7.0%	12.7%	0.9%	7.2%	9.2%	8.8%
Guatemala	Low-Mid	7885.07	10152.08	28.8%	11.3%	8.2%	14.9%	1.1%	8.6%	11.0%	10.6%
Honduras	Low-Mid	5476.92	6790.50	24.0%	9.7%	7.1%	12.9%	0.9%	7.3%	9.4%	8.9%
Nicaragua	Low-Mid	5778.65	6762.57	17.0%	7.1%	5.3%	9.8%	0.7%	5.4%	6.9%	6.4%
**Central America**	** **	** **	** **	**22.7%**	**7.9%**	**6.5%**	**11.8%**	**0.8%**	**6.8%**	**8.6%**	**8.0%**
Canada	Hi	48389.86	55097.00	13.9%	3.8%	4.4%	8.2%	0.6%	4.5%	5.4%	4.0%
United States	Hi	59628.62	75401.22	26.5%	6.7%	7.7%	14.0%	1.0%	8.0%	7.7%	7.3%
Mexico	Up-Mid	19817.61	22328.21	12.7%	4.9%	4.1%	7.6%	0.5%	4.2%	5.3%	4.8%
**North America (Mex excluded)**	** **	** **	** **	**17.7%**	**5.1%**	**5.4%**	**9.9%**	**0.7%**	**5.5%**	**6.2%**	**5.4%**
Argentina	Hi	23369.99	23592.87	1.0%	0.3%	0.3%	0.6%	0.0%	0.3%	0.4%	0.4%
Chile	Hi	23822.12	28380.79	19.1%	5.1%	5.9%	10.8%	0.8%	6.0%	7.7%	7.2%
Uruguay	Hi	22431.88	26607.68	18.6%	5.3%	5.7%	10.5%	0.8%	5.9%	7.5%	7.0%
Brazil	Up-Mid	14748.41	17259.89	17.0%	7.1%	5.3%	9.8%	0.7%	5.4%	6.9%	6.4%
Colombia	Up-Mid	14750.20	18835.73	27.7%	10.9%	8.0%	14.5%	1.0%	8.3%	10.6%	10.2%
Ecuador	Up-Mid	11649.93	12607.56	8.2%	3.6%	2.8%	5.1%	0.4%	2.8%	3.5%	3.1%
Guyana	Up-Mid	11815.23	56006.54	374.0%	43.1%	32.1%	50.3%	1.2%	40.9%	56.6%	88.7%
Paraguay	Up-Mid	12207.68	16123.30	32.1%	12.4%	9.0%	16.2%	1.1%	9.4%	12.0%	11.8%
Peru	Up-Mid	12457.41	15900.97	27.6%	10.9%	8.0%	14.4%	1.0%	8.3%	10.6%	10.2%
Suriname	Up-Mid	18326.83	16324.22	-10.9%	-5.4%	-4.3%	-8.4%	-0.6%	-4.2%	-5.4%	-4.3%
Bolivia	Low-Mid	8348.02	10416.83	24.8%	9.9%	7.3%	13.3%	0.9%	7.5%	9.7%	9.2%
**South America (Guyana excluded)**		** **	** **	**16.5%**	**6.0%**	**4.8%**	**8.7%**	**0.6%**	**5.0%**	**6.4%**	**6.1%**

Notes

* World Bank classification

** IMF

*** Estimation based on IMF’s projections

Source: own elaboration

The differences in the expected GDP growth rates explain much of the countries disparities in the expected progress in each health theme. The projected progress in the seven health-related themes for the subregions ranges from 0.6% in non-communicable diseases for South America to 11.8% for infectious diseases in Central America. Suriname, Haiti and Belize are the only countries in the sample for which the IMF predicts a contraction of the GDPpc in the period. Hence, a worsening in the SDGs in all three countries. On the opposite side, the IMF expects Guyana to triple its GDP on the period, something reflected in the skyrocketing progress the country is expected to see in most health-related themes. Overall, Central America shows the best prospects followed by South America.

The results also underscore the expected progress in the theme of infectious diseases in the countries and subregions of the continent. The thematic group comprises 5 indicators, and although Tuberculosis (3.3.2) and Hepatitis B (3.3.4) incidence is expected to display consistent reduction in the period, much of this outstanding result is explained by the expected progress in NTDs (3.3.5) and Malaria incidence (3.3.3).

Environmental risks and health systems and coverage are also expected to receive a big push due to improvements in GDPpc, with increments ranging from 5.4% to 8.6% on the sub-regions average. On the opposite side, non-communicable diseases will likely improve at a much slower pace. This is explained by the controversial effects of the economic growth on relevant risk factors, which was captured in the GDPpc parameters in the estimated evolution curves. Even in countries set to grow at a fast pace, like Guyana and the Dominican Republic, the average improvement in the theme is of only 1.2% and 1.8%, respectively.

## 4. Discussion

The global development curves presented in the last section revealed interesting patterns of the evolution of the health-related SDGs indicators. While the majority of the health topics seems to evolve–in a greater or lesser degree–pari passu with the development process summarized in the GDPpc, regional factors, the level of inequality and the public investment in health also proved important in the determination of some indicators. The Gini index and the health expenditure are particularly relevant in the themes of Newborn and child health, Infectious diseases and Injuries and Violence. The level of health expenditure is also significant in the explanation of environmental risks. Regional patterns are more evident in the theme of infectious diseases, but also in indicators of child nutrition, alcohol consumption, tobacco use, sanitation facilities and adolescent birth rate. South-East Asia and Europe were the regions with more singular characteristics in the health-related topics, as demonstrated by the significance of the regional dummies (see Table 2). A closer look at how the specific health issues are treated in these regions may reveal important lessons to the rest of the world.

The slopes of the global development curves present a clear indicative of the topics with easier/harder progress. The progress in infant and maternal mortality and in the themes of health system and coverage and infectious diseases–with the notable exception of HIV infections, which is highly associated with regional factors–are strongly associated with the progress in the GDPpc. On the other side, non-communicable diseases, injuries and violence and some indicators of environmental risks show a much lower sensitivity to increments in the GDPpc, requiring thus more specific efforts for the achievement of the global targets.

Indeed, some of the results represent a warning call for public policymakers in the region. For instance, the positive association between increases in the GDPpc and the consumption of alcohol (3.5.2), tobacco use (3.a.1) and overweight among children (2.2.2b) require an articulated set of policies to inhibit the natural path of these morbidity factors. On the other hand, the non-significant results for all vaccination indicators are an important indicative of the relevance of local and political factors in the determination of the phenomenon, which should be investigated with studies focusing on countries individually, given the multiplicity of factors involved in the phenomena.

The lack of data for indicators 3.7.1 and 3.7.2 –access to contraception and adolescent birth rate–raise worries on the transparency of women’s reproductive and sexual rights in the region, one of the most inequitable interventions worldwide [19]. Likewise, the lack of data on indicators 2.2.1 and 2.2.2 is a call to action to collect timely and quality data on child nutrition in the region. Before the COVID-19 crisis, the rates of maternal and child undernutrition (underweight, stunted, and micronutrients deficiency) were slowly declining in low-income and middle-income countries (LMICs), while middle-income countries were experiencing marked decreases [20, 21]. The unprecedented disruptions in global health, economic, and food systems have contributed to decreases in the coverage of routine health services, sharp increases in household food insecurity, and anticipated setbacks to global nutrition targets [20]. Socioeconomic inequalities persist as a significant distal determinant of undernutrition for women and children [22]. Since 2013, the range of evidence-based interventions within the field has grown significantly, pointing to successful experiences and the need for integrated correctional programs that ensure food security [20].

The analysis of the health gaps in the Americas revealed great intraregional disparities in the health-related topics, despite the cultural similarities and geographical proximity of the countries. Overall, the positive highlights were the environmental risk indicators. These were better than expected according to the global counterfactual. On the other hand, the biggest gaps in the region were in the themes of violence, especially that measured by the homicide indicator (16.1.1) and some infectious diseases, as the NTDs (3.3.5). On the bright side, these are amongst the areas with greater potential gains from the process of development.

The gap index offers a glimpse of the main health challenges in the region, but without a proper assessment of the trends in the evolution of the indicators, not much can be said about the necessary additional effort required by these countries to reach the SDGs by 2030. In fact, due to the characteristics of each health topic, in some cases, advances in indicators in green zone (above expectations) can be costlier than in those which the country is still lagging behind (red zone). This is the case for the curves for which the GDPpc slope is close to zero (or statistically indistinct from it).

The significance of the GDPpc slope in most topics reinforces the role of economic growth for the achievement of the 2030 health-related targets. Countries with good economic prospects are likely to enjoy greater improvement in most indicators. If the conditions detailed in the Methods and Data section hold true, the forecasts can be read as a measure of the ‘inertial progress’, i.e., the hypothetical future situation of these countries if the international trends apply to the country and no specific policies or complementary effort are implemented to accelerate the achievement of targets.

The results show that the countries in the region will likely see an above average progress in the themes of Infectious diseases, Environmental risks and Health systems and coverage. Considering the key position of the latter as a determinant of all other health topics, one should highlight the potential cross-impact of improvements of health infrastructure, access and coverage on all morbimortality indicators.

The COVID-19 pandemic has underlined the need to accelerate progress towards universal health coverage and universal access to key health infrastructure. Progress in the theme will likely require integrated delivery care system and access to high-quality and effective health services–including those designed to promote health, prevent illness, and to provide treatment, rehabilitation and palliative care–without exposing users to financial hardship [23]. Improvements in health system and coverage likely improves all other health indicators. Before the pandemic, service coverage was slowing improving worldwide. Progress has been more marked in low income countries, starting from a lower base and mainly driven by interventions for infectious diseases and, to a lesser extent, for reproductive, maternal, newborn and child health services [14], corroborating with results predicted in this study. The investment in creating universal health systems, resilient to health and economic crises will be a strong differential to all regions for achieving the 2030 Agenda goals and targets.

On the opposite side, non-communicable diseases will likely improve at a much slower pace. This is explained by the controversial effects of the economic growth on relevant risk factors. In many countries, increases of income are followed by increases in alcohol and tobacco consumption, which affects these two indicators themselves and also the prevalence of other non-communicable diseases. Recent studies show that late detection and postponing treatments of cardiovascular diseases, cancer, diabetes, and chronic respiratory diseases are amongst the most impacted by the COVID-19 effect on the reduction in population mobility and the decrease in health services capacity [24–26]. In some countries, suicides and mental health conditions have also deteriorated due to the pandemic and its socioeconomic effects [27].

The full epidemiological, socioeconomic, and environmental impacts of the COVID-19 outbreak are still unknown and will potentially change the current and future situation of the countries in the region. Some health-related indicators figure amongst the most affected by the disease, either directly–by its impact on the population morbimortality–and/or indirectly–by the influence of the socioeconomic determinants of health. Although the GDP forecasts include the latest assessments of economic impact of the pandemic outbreak, as of June 2021, the delayed vaccination schedule and the emergence of new variants of the virus can still affect the economic recovery of the region. Even though the latest data available cannot show the impact of the pandemics on the health-related indicators, the analysis of the COVID-19 responses around the world offer a reminder that efforts should be made to strengthen local-level public health responses, infrastructure and health access, which proved key also to the economic resilience of countries [28, 29].

Naturally, the approach in this study has important limitations and do not cancel the need of country-specific studies. Indeed, the power of these global curves to represent the country-specific phenomenon is closely connected to the importance of transnational factors in their determination. Moreover, the empirical evidence of the association between the growth of GDPpc and improvements in the health-related indicators does not imply that the first is a determinant of these. Hence, deliberate efforts to boost the country’s GDP might not be the best nor the shortest path for achieving of health-related goals. The approach, notwithstanding, provides critical input for improving the allocation of public resources.

## 5. Conclusions and recommendations

This paper sought to assess the pre-pandemics health gaps in the Americas and predict the evolution of the health-related indicators for the SDGs in the region using a methodology that takes advantage of the increasing availability of comparable international data. The approach proved successful for the analysis of the vast majority of topics, revealing important characteristics of the global evolution of the health indicators. The study also pointed out important intra-regional disparities in the indicators, which is likely to have increased with the COVID-19 pandemic.

Ultimately the results in this paper indicate that the ability of the countries in the region to fund the post COVID-19 recovery will likely determine much of the success or failure in the SDGs. While high-income countries have borrowed heavily in response to the pandemic, the poorest countries in the globe and region have been unable to do so. Concomitantly, many developed countries are currently implementing massive investment programs in several areas, from economic conversion to health structure, whereas recovery in most developing countries is constrained by strict austerity policies. This, of course, can increase inequalities worldwide. TWI2050 (2020) has shown that billions were left behind and the projected progress in the health-related measures in America clearly reflects the same [30]. With less than ten years left for countries to achieve their 2030 commitments, leaving no one behind will require innovative and cross-sectoral coordination [31]. As the COVID-19 crisis has shown, countries with effective social protection systems and universal health coverage are best equipped to respond not only to the actual but also future crises [1]. A considerable list of challenges lay ahead, such as: insecurity, conflicts and disease outbreaks; lack of sustained political commitment and inappropriate monitoring and evaluation structures; inappropriate and unsustainable financing models; insufficient health workforce recruitment, employment and retention; missing support of physicians and their professional organizations; inadequately addressing the needs of the community and not giving attention to gender equity [14, 32].

This study thus indicates the need of more and more concerted efforts to meet the SDGs’ targets, especially in face of the COVID-19 impact on the epidemiological and socioeconomic prospects of these countries. The high level of aggregation of the analysis, based on the country data, likely masks an even higher effort needed. In fact, the effectiveness of the 2030 Agenda’s principle of leaving no one behind requires a closer look to regional and local inequalities. For countries at an intermediate level of development, progress in the health-related SDGs can still be made by increasing access to health services, but the real challenges ahead can only be addressed by reducing inequalities.

## Supporting information

S1 Appendix Health in 2030: American countries, forecast (Tables A1a, A1b and A1c). (PDF)

S1 Database (XLSX)
