# Supplementary material for: The sustainable health Agenda in the Americas: Pre-pandemic gaps and 2030 estimates of the SDGs indicators
Source: PLoS One. 2022 Jun 21;17(6):e0270301. doi: 10.1371/journal.pone.0270301 (PMC9212141; doi:10.1371/journal.pone.0270301)
Supplement: S1 Appendix — (PDF) [file pone.0270301.s001.pdf]

Table A1a - Health in 2030: The Caribbean countries, forecasts

	Indicator	The Caribbean												
		**Average**	Antigua and Barbuda	Bahamas	Barbados	Dominica	Dominican Republic	Grenada	Haiti	Jamaica	Saint Kitts and Nevis	Saint Lucia	Saint Vincent and the Grenadines	Trinidad and Tobago
311	Maternal mortality ratio (per 100 000 live births)	**86.46**	-	77.40	25.60	-	62.77	-	60.83	22.31	80.20	43.11	35.84	370.05
312	Births attended by skilled health personnel (%)	**94.96**	100.00	99.43	99.69	100.00	100.00	100.00	100.00	100.00	98.93	100.00	100.00	41.44
322	Under-five mortality rate (probability of dying by age 5 per 1000 live births)	**12.00**	3.67	4.08	7.94	7.99	17.08	20.75	14.22	9.03	10.13	11.52	8.09	29.46
321	Neonatal mortality rate (per 1000 live births)	**.**	6.92	7.32	12.03	12.07	26.79	24.43	19.36	13.35	13.97	15.04	13.02	75.90
331	New HIV infections (per 1000 uninfected population)	**0.47**	-	0.38	0.57	-	0.24	-	0.24	-	0.67	-	-	0.73
332	Incidence of tuberculosis (per 100 000 population per year)	**24.84**	2.83	19.34	0.39	2.07	17.30	5.96	39.98	4.54	4.46	5.90	4.69	190.58
333	Malaria incidence (per 1 000 population at risk)	**1.81**	-	-	-	-	-	-	0.06	-	-	-	-	3.57
334	Reported number of people requiring interventions against NTDs	**0.48**	0.36	0.30	0.33	0.35	0.43	0.33	0.26	0.42	0.15	0.37	0.37	2.08
335	Hepatitis B surface antigen (HBsAg) prevalence among children under 5 years (%)	**7.82**	0.00	0.04	0.01	0.00	0.19	0.05	8.68	0.00	2.85	0.24	0.00	81.76
341	Probability (%) of dying between age 30 and exact age 70 from any of cardiovascular disease, cancer, diabetes, or chronic respiratory disease	**19.18**	21.88	15.38	15.96	-	21.37	-	16.57	20.21	14.24	18.23	21.07	26.87
342	Crude suicide rates (per 100 000 population)	**5.09**	0.64	1.74	0.74	-	13.59	-	8.92	1.34	2.10	7.61	2.42	11.78
352	Total (recorded+unrecorded) alcohol per capita (15+) consumption	**8.02**	7.26	4.46	9.80	9.84	8.41	9.06	8.09	10.01	4.37	10.32	8.95	5.73
361	Estimated road traffic death rate (per 100 000 population)	**15.41**	7.71	-	5.53	-	12.09	10.21	31.18	8.86	13.25	34.46	-	-
371	Women of reproductive age (aged 15-49 years) who have their need for family planning satisfied with modern methods (%)	**42.26**	-	-	-	-	-	-	-	-	-	-	-	42.26
372	Adolescent birth rate (per 1000 women aged 15-19 years)	**-**	-	-	-	-	-	-	-	-	-	-	-	-
381	Universal health coverage (UHC) service coverage index	**76.13**	79.00	74.00	79.95	-	73.22	-	100.00	82.56	69.22	73.23	83.29	46.81
391	Ambient and household air pollution attributable death rate (per 100 000 population)	**45.99**	26.83	21.68	55.66	-	44.93	-	31.57	40.39	30.54	36.21	43.35	128.74
392	Mortality rate attributed to exposure to unsafe WASH services (per 100 000 population)	**2.88**	0.09	0.10	0.19	-	0.10	-	1.26	0.23	0.52	0.52	0.96	24.79
393	Mortality rate attributed to unintentional poisoning (per 100 000 population)	**0.47**	0.37	0.10	0.19	-	0.20	-	0.28	0.30	0.18	0.18	0.16	2.72
221	Children aged <5 years stunted (% height-for-age <-2 SD)	**22.40**	-	-	-	-	-	-	-	-	-	-	-	22.40
222a	Children aged <5 years wasted (% weight-for-height <-2 SD)	**3.76**	-	-	-	-	-	-	-	-	-	-	-	3.76
222b	Children aged <5 years overweight (% weight-for-height >+2 SD)	**3.37**	-	-	-	-	-	-	-	-	-	-	-	3.37
222c	Children aged <5 years underweight (% weight-for-age <-2 SD) (%)	**9.77**	-	-	-	-	-	-	-	-	-	-	-	9.77
3a	Age-standardized prevalence of current tobacco smoking among persons aged 15 years and older	**12.67**	-	11.78	8.17	-	-	-	13.46	-	16.89	-	-	13.02
3c1a	Medical doctors (per 10 000 population)	**19.37**	29.99	19.93	26.04	27.93	26.78	13.51	22.18	17.02	8.00	-	-	2.29
3c1b	Nursing and midwifery personnel (per 10 000 population)	**33.28**	32.84	31.96	62.05	42.46	35.16	68.00	3.88	34.89	14.88	-	-	6.69
3c1c	Dentists (per 10 000 population)	**2.19**	-	2.65	3.21	4.04	3.61	0.83	2.89	1.83	0.43	-	-	0.21
3c1d	Pharmacists (per 10 000 population)	**3.44**	-	-	-	-	5.07	-	-	8.12	0.27	-	-	0.29
3d1	IHR capacity and health emergency preparedness (Average of 13 International Health Regulations core capacity scores, SPAR version)	**65.43**	70.66	65.63	88.96	53.72	66.01	65.03	62.56	66.78	80.51	65.43	49.01	50.91
611	Population using at least basic drinking-water services (%)	**95.67**	99.09	98.29	99.18	-	97.08	100.00	100.00	100.00	95.19	100.00	99.96	63.57
621	Population using at least basic sanitation services (%)	**87.08**	91.05	93.09	97.84	-	92.12	85.60	96.21	94.21	88.22	94.56	94.36	30.67
712	Proportion of population with primary reliance on clean fuels and technologies	**91.03**	100.00	97.06	98.34	100.00	95.22	100.00	100.00	100.00	96.83	100.00	100.00	4.90
1162	Concentrations of fine particulate matter (PM2.5)	**18.03**	17.84	18.94	22.29	12.16	22.39	18.34	12.79	21.41	13.47	20.99	20.95	14.74
1611	Estimates of rates of homicides per 100 000 population	**19.28**	1.69	29.13	9.91	-	42.20	-	12.97	5.58	36.77	14.52	11.67	28.40

Notes: Indicators 3.b.1a,b,c and 17.19.2 omitted due to the lack of statistical significance of the estimations.

Source: own elaboration

Table A1b - Health in 2030: Central and North American countries, forecasts

	Indicator	Central America								North America			
		**Average**	Belize	Costa Rica	El Salvador	Guatemala	Honduras	Nicaragua	Panama	**Average (Mexico excluded)**	Canada	United States	Mexico
311	Maternal mortality ratio (per 100 000 live births)	**68.52**	28.31	20.37	72.66	45.58	71.87	108.59	132.25	**8.95**	6.31	11.60	34.54
312	Births attended by skilled health personnel (%)	**88.97**	92.07	98.70	94.20	100.00	71.05	75.68	91.08	**98.50**	97.90	99.10	98.92
322	Under-five mortality rate (probability of dying by age 5 per 1000 live births)	**7.77**	9.29	4.99	7.29	6.29	11.10	8.90	6.54	**3.17**	3.22	3.12	6.97
321	Neonatal mortality rate (per 1000 live births)	**14.46**	15.09	7.32	12.49	12.41	22.80	15.59	15.52	**5.03**	4.63	5.43	12.67
331	New HIV infections (per 1000 uninfected population)	**0.30**	0.93	0.23	0.42	0.20	0.14	0.10	0.07	**-**	-	-	0.12
332	Incidence of tuberculosis (per 100 000 population per year)	**35.56**	33.19	9.07	47.38	55.36	22.56	36.34	45.03	**4.00**	5.12	2.88	20.64
333	Malaria incidence (per 1 000 population at risk)	**0.47**	0.03	0.00	0.12	0.00	0.36	0.37	2.40	**-**	-	-	0.19
334	Reported number of people requiring interventions against NTDs	**0.39**	1.50	0.15	0.19	0.51	0.04	0.22	0.13	**0.50**	0.97	0.04	0.04
335	Hepatitis B surface antigen (HBsAg) prevalence among children under 5 years (%)	**9.86**	0.07	0.13	3.04	7.43	28.33	20.42	9.64	**0.00**	0.00	0.01	140.00
341	Probability (%) of dying between age 30 and exact age 70 from any of cardiovascular disease, cancer, diabetes, or chronic respiratory disease	**14.17**	22.33	10.78	12.01	13.27	13.92	13.21	13.71	**11.56**	9.56	13.56	15.30
342	Crude suicide rates (per 100 000 population)	**6.70**	4.76	7.54	3.87	13.33	2.77	2.74	11.86	**13.36**	12.21	14.52	5.24
352	Total (recorded+unrecorded) alcohol per capita (15+) consumption	**5.27**	6.67	5.19	8.72	3.95	2.59	4.27	5.46	**9.90**	9.26	10.53	6.74
361	Estimated road traffic death rate (per 100 000 population)	**18.45**	28.38	15.86	13.40	21.27	15.78	15.99	-	**8.74**	5.65	11.83	12.79
371	Women of reproductive age (aged 15-49 years) who have their need for family planning satisfied with modern methods (%)	**70.39**	65.43	-	-	-	75.35	-	-	**77.20**	-	77.20	83.15
372	Adolescent birth rate (per 1000 women aged 15-19 years)	**73.50**	-	-	73.50	-	-	-	-	**20.18**	-	20.18	-
381	Universal health coverage (UHC) service coverage index	**77.29**	64.41	90.62	77.50	86.86	66.21	75.53	79.91	**86.00**	88.50	83.50	82.25
391	Ambient and household air pollution attributable death rate (per 100 000 population)	**34.90**	35.17	24.63	23.17	38.93	45.67	36.11	40.64	**18.19**	14.32	22.06	31.62
392	Mortality rate attributed to exposure to unsafe WASH services (per 100 000 population)	**2.02**	1.02	0.68	1.34	1.59	4.80	2.86	1.86	**0.25**	0.35	0.16	0.97
393	Mortality rate attributed to unintentional poisoning (per 100 000 population)	**0.44**	0.50	0.25	0.32	0.17	0.96	0.34	0.54	**0.52**	0.27	0.76	0.37
221	Children aged <5 years stunted (% height-for-age <-2 SD)	**27.65**	15.12	-	-	-	40.17	-	-	**3.04**	-	3.04	10.43
3a	Age-standardized prevalence of current tobacco smoking among persons aged 15 years and older	**1.27**	1.81	-	-	-	0.72	-	-	**17.93**	14.21	21.65	14.12
3b1a	Diphtheria tetanus toxoid and pertussis (DTP3) immunization coverage among 1-year	**6.25**	7.28	-	-	-	5.22	-	-	**93.52**	91.36	95.68	97.35
3b1b	Measles-containing-vaccine second-dose (MCV2) immunization coverage by the nation	**7.45**	4.65	-	-	-	10.25	-	-	**91.49**	87.00	95.98	99.04
3b1c	Pneumoccocal conjugate vaccines (PCV3) immunization coverage among 1-year-olds	**9.48**	-	11.75	6.10	10.59	-	-	-	**85.78**	79.20	92.37	91.18
3c1a	Medical doctors (per 10 000 population)	**11.60**	11.16	-	19.54	18.12	4.22	5.34	11.19	**29.25**	28.10	30.39	24.40
3c1b	Nursing and midwifery personnel (per 10 000 population)	**16.62**	16.84	-	16.22	24.98	10.57	14.19	16.90	**99.60**	104.48	94.72	29.33
3c1c	Dentists (per 10 000 population)	**1.22**	1.53	0.12	3.41	-	0.12	1.72	0.45	**6.94**	6.82	7.05	1.22
3c1d	Pharmacists (per 10 000 population)	**4.76**	6.72	-	-	7.52	0.05	-	-	**11.51**	10.78	12.24	-
3d1	IHR capacity and health emergency preparedness (Average of 13 International Health Regulations core capacity scores, SPAR version)	**72.19**	43.97	82.27	72.64	89.67	68.17	65.94	82.63	**98.31**	100.00	96.62	93.80
611	Population using at least basic drinking-water services (%)	**95.32**	96.76	100.00	95.56	96.60	98.36	95.62	84.34	**99.00**	99.00	99.00	100.00
621	Population using at least basic sanitation services (%)	**86.33**	86.66	100.00	84.41	96.68	72.02	85.07	79.49	**100.00**	100.00	100.00	92.09
712	Proportion of population with primary reliance on clean fuels and technologies	**77.96**	84.39	100.00	100.00	95.43	51.31	58.70	55.88	**97.50**	100.00	95.00	90.43
1162	Concentrations of fine particulate matter (PM2.5)	**19.44**	20.92	16.38	11.71	23.42	23.74	21.15	18.78	**7.05**	6.63	7.47	20.71
1611	Estimates of rates of homicides per 100 000 population	**26.41**	29.91	8.95	17.39	41.18	23.72	50.96	12.78	**3.47**	1.40	5.54	15.76

Notes: Indicators 3.b.1a,b,c and 17.19.2 omitted due to the lack of statistical significance of the estimations.

Source: own elaboration

Table A1c - Health in 2030: South American countries, forecasts

Indicator		South America											
		**Average**	Argentina	Chile	Uruguay	Brazil	Colombia	Ecuador	Guyana	Paraguay	Peru	Suriname	Bolivia
311	Maternal mortality ratio (per 100 000 live births)	**73.21**	51.61	19.12	13.08	38.79	52.62	60.08	65.85	105.63	55.93	170.05	172.53
312	Births attended by skilled health personnel (%)	**93.84**	93.99	99.70	100.00	100.00	100.00	97.20	-	100.00	95.50	79.04	72.97
322	Under-five mortality rate (probability of dying by age 5 per 1000 live births)	**7.94**	6.26	4.55	4.21	8.18	6.95	7.27	6.32	9.35	6.40	11.53	16.35
321	Neonatal mortality rate (per 1000 live births)	**13.98**	10.81	6.55	7.35	13.55	12.21	13.79	8.00	16.88	12.51	22.45	29.65
331	New HIV infections (per 1000 uninfected population)	**0.28**	0.15	0.34	0.19	0.24	-	0.12	0.76	0.21	0.09	0.55	0.14
332	Incidence of tuberculosis (per 100 000 population per year)	**45.90**	25.90	15.52	27.99	40.02	28.97	39.38	48.13	37.80	106.24	30.75	104.18
333	Malaria incidence (per 1 000 population at risk)	**2.26**	0.00	-	-	3.21	5.82	1.91	2.93	0.00	4.38	0.94	1.19
334	Reported number of people requiring interventions against NTDs	**0.27**	0.01	0.26	0.32	0.06	0.19	0.31	0.44	0.57	0.21	0.38	0.18
335	Hepatitis B surface antigen (HBsAg) prevalence among children under 5 years (%)	**16.05**	0.29	0.00	0.00	85.80	27.54	17.43	1.19	8.57	20.86	0.65	14.24
341	Probability (%) of dying between age 30 and exact age 70 from any of cardiovascular disease, cancer, diabetes, or chronic respiratory disease	**15.89**	15.91	11.87	16.14	16.10	14.84	12.92	20.06	16.33	11.89	22.44	16.30
342	Crude suicide rates (per 100 000 population)	**11.92**	8.98	10.41	18.32	6.32	7.41	8.23	21.97	9.22	4.69	23.80	11.81
352	Total (recorded+unrecorded) alcohol per capita (15+) consumption	**7.71**	9.83	9.81	11.38	8.19	6.25	4.51	10.16	7.84	6.79	4.92	5.14
361	Estimated road traffic death rate (per 100 000 population)	**16.24**	13.97	12.07	12.95	19.09	17.61	20.96	17.98	21.46	12.85	14.84	14.82
371	Women of reproductive age (aged 15-49 years) who have their need for family planning satisfied with modern methods (%)	**80.23**	-	-	-	-	98.30	-	-	91.14	75.05	-	56.42
372	Adolescent birth rate (per 1000 women aged 15-19 years)	**56.58**	65.24	-	51.69	56.86	-	-	-	-	44.52	-	64.60
381	Universal health coverage (UHC) service coverage index	**83.05**	76.48	76.37	89.02	87.12	89.37	80.61	100.00	82.37	90.52	65.30	76.43
391	Ambient and household air pollution attributable death rate (per 100 000 population)	**38.54**	36.87	32.87	30.10	29.30	31.15	21.39	43.49	41.63	53.14	53.16	50.80
392	Mortality rate attributed to exposure to unsafe WASH services (per 100 000 population)	**1.12**	0.40	0.17	0.33	0.84	0.61	0.55	0.67	1.11	1.00	2.27	4.41
393	Mortality rate attributed to unintentional poisoning (per 100 000 population)	**0.52**	0.65	0.18	0.36	0.18	0.34	0.57	0.24	0.29	0.76	0.43	1.76
221	Children aged <5 years stunted (% height-for-age <-2 SD)	**10.19**	-	-	-	-	-	-	-	4.74	11.73	-	14.11
3a	Age-standardized prevalence of current tobacco smoking among persons aged 15 years and older	**18.12**	21.99	37.58	16.86	13.89	8.99	7.17	-	13.12	-	25.34	-
3b1a	Diphtheria tetanus toxoid and pertussis (DTP3) immunization coverage among 1-year	**90.70**	89.03	94.50	95.49	89.42	92.18	84.20	101.68	92.78	86.64	85.70	86.08
3b1b	Measles-containing-vaccine second-dose (MCV2) immunization coverage by the nation	**79.56**	88.57	89.89	93.41	59.83	89.93	68.98	107.37	86.62	67.45	43.55	-
3b1c	Pneumoccocal conjugate vaccines (PCV3) immunization coverage among 1-year-olds	**87.37**	78.98	92.03	93.53	83.62	89.36	83.81	90.32	95.23	82.41	-	84.45
3c1a	Medical doctors (per 10 000 population)	**22.70**	39.86	12.17	50.45	23.93	23.72	21.76	23.07	9.71	15.00	11.34	18.73
3c1b	Nursing and midwifery personnel (per 10 000 population)	**26.65**	25.91	9.28	44.22	83.88	13.37	19.09	26.42	8.85	15.02	38.96	8.10
3c1c	Dentists (per 10 000 population)	**5.90**	-	1.78	14.95	13.63	11.16	4.46	0.93	1.56	2.09	-	2.56
3c1d	Pharmacists (per 10 000 population)	**1.94**	-	-	-	7.51	-	0.44	0.24	0.38	-	0.41	2.63
3d1	IHR capacity and health emergency preparedness (Average of 13 International Health Regulations core capacity scores, SPAR version)	**80.05**	73.28	78.67	84.96	94.98	82.99	81.31	91.73	76.03	70.84	71.59	74.13
611	Population using at least basic drinking-water services (%)	**97.99**	100.00	100.00	100.00	99.77	100.00	94.33	100.00	100.00	94.03	93.05	96.77
621	Population using at least basic sanitation services (%)	**88.84**	95.26	100.00	100.00	89.95	90.08	87.96	100.00	98.53	82.56	76.43	56.46
712	Proportion of population with primary reliance on clean fuels and technologies	**93.52**	95.47	100.00	100.00	100.00	100.00	98.98	100.00	75.68	84.57	84.28	89.75
1162	Concentrations of fine particulate matter (PM2.5)	**17.74**	11.69	22.80	8.59	11.66	16.88	15.41	19.20	11.46	28.47	26.03	22.91
1611	Estimates of rates of homicides per 100 000 population	**12.98**	6.22	4.11	6.78	28.97	38.51	8.69	8.56	7.54	10.24	10.61	12.50

Notes: Indicators 3.b.1a,b,c and 17.19.2 omitted due to the lack of statistical significance of the estimations.

Source: own elaboration
